# Hydatid Cyst in Pregnancy—A Diagnostic and Therapeutic Dilemma: Study Case Report

**DOI:** 10.3390/jcm14145073

**Published:** 2025-07-17

**Authors:** Liliana Steriu, Ionut Eduard Iordache, Antonia Bisinicu, Bianca Andreea Steriu, Gabriela Baltatescu, Andreea Nelson Twakor, Eugen Dumitru, Vlad Tica

**Affiliations:** 1 Obstetrics & Gynaecology Department, Faculty of Medicine, “Ovidius” University, 900470 Constanta, Romania; lilianasteriu@yahoo.com (L.S.); vtica@eeirh.org (V.T.); 2 Department of General Surgery, “Sf. Apostol Andrei” Emergency County Hospital, 145 Tomis Blvd., 900591 Constanta, Romania; 3 Faculty of Medicine and Pharmacy Constanta, Ovidius University, 900470 Constanta, Romania; antonia.bisinicu@yahoo.com (A.B.); bianca.steriu@cnmbct.ro (B.A.S.); 4 Center for Research and Development of the Morphological and Genetic Studies of Malignant Pathology—CEDMOG, Faculty of Medicine and Pharmacy Constanta, Ovidius University, 900470 Constanta, Romania; gabrielabaltatescu@yahoo.com; 5 Internal Medicine Department, “Sf. Apostol Andrei” Emergency County Hospital, 145 Tomis Blvd., 900591 Constanta, Romania; andreea.purcaru@365.univ-ovidius.ro; 6 Gastroenterology Department, “Sf. Apostol Andrei” Emergency County Hospital, 145 Tomis Blvd., 900591 Constanta, Romania; eugen.dumitru@univ-ovidius.ro

**Keywords:** hydatid cyst, Echinococcus granulosus, pregnancy, laparoscopic surgery, multidisciplinary care, PAIR

## Abstract

**Background:** Hydatid disease, caused by the larval form of Echinococcus granulosus, is a rare but potentially life-threatening condition during pregnancy, with an estimated incidence of 1 in 20,000 to 30,000 gestations. Physiological immunosuppression and increased placental steroid levels during pregnancy may promote cyst growth, elevating the risk of rupture, which can result in anaphylactic shock, sepsis, or widespread peritoneal dissemination. Diagnostic imaging, particularly ultrasonography, plays a central role in detection, while treatment decisions are complicated by the lack of standardized guidelines and the need to balance maternal–fetal safety. **Methods**: This case report describes a 29-year-old pregnant woman at 22 weeks’ gestation who was incidentally diagnosed with two large hepatic hydatid cysts during a routine ultrasound. **Results:** Given the high rupture risk, she underwent successful laparoscopic surgery in the second trimester, followed by careful monitoring and elective cesarean delivery at term. A third retroperitoneal cyst, initially managed conservatively, was excised postpartum. **Conclusions:** This case highlights the critical importance of individualized, multidisciplinary management in achieving favorable maternal and neonatal outcomes in complex presentations of hydatid disease during pregnancy.

## 1. Introduction

Hydatid cystic disease, often known as cystic echinococcosis (CE), is a zoonotic infectious disease caused by *Echinococcus granulosus* larvae [1]. Dogs are the primary hosts for this 3–6 mm taeniid-type tapeworm. Infected dogs excrete tapeworm eggs, which herbivorous intermediate hosts like sheep and cattle eat to perpetuate the *E. granulosus* lifecycle [2]. After ingestion, the parasite transforms into the digestive system, penetrating the mucosal lining and spreading hematogenously to highly vascularized organs as the liver (60%) and lungs (30%) [3].

Humans become unintentional intermediate hosts by touching diseased dogs or eating contaminated food or drink. Once inside the host, larval cysts develop over months to years, generally asymptomatic until they become large or cause difficulties [4].

**Figure 1:**
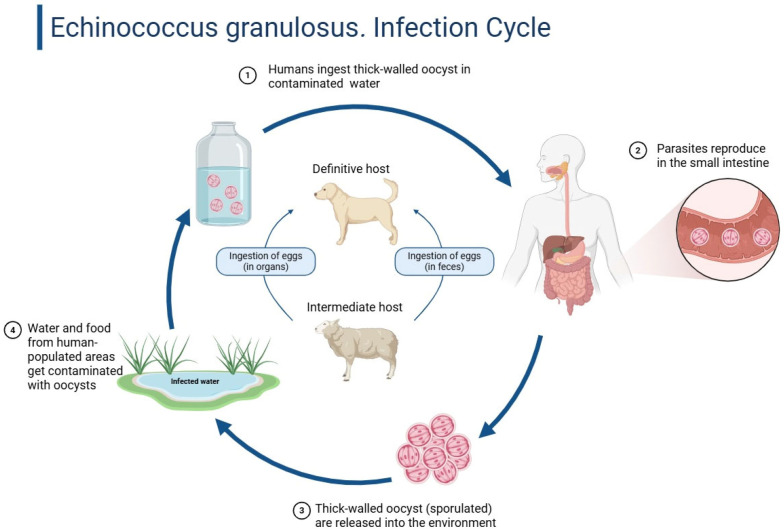
Echinococcus granulosus: infection cycle diagram. The cycle begins with humans (accidental hosts) ingesting thick-walled oocysts through contaminated water (Step 1). Once inside the human host, the parasites migrate to the small intestine and reproduce (Step 2). The definitive host, typically a dog, ingests the parasite’s eggs present in the organs of intermediate hosts like sheep (Step 3). These eggs develop into adult worms in the definitive host’s intestine and thick-walled oocysts are released into the environment via feces (Step 4). The environment becomes contaminated and water or vegetation exposed to these oocysts can then infect new hosts, completing the cycle. Created with Biorender [6].

Hydatid illness is endemic in the Mediterranean, Middle East, South America and portions of Africa, although its pregnancy incidence is extremely low, estimated from 1 in 20,000 to 1 in 30,000 [5]. Prenatal imaging may accidentally diagnose the disease, which is often asymptomatic. Pregnancy-induced immune modulation, particularly cell-mediated immunity reduction, can promote cyst formation. This increases the risk of mechanical compression, rupture and secondary infection, compromising maternal and fetal health [4]. Figure 1 below shows the infection cycle of echinococcus granulosus.

For standardized assessment and management, two major classification systems are widely used: the Gharbi classification, which is based on ultrasound imaging features and divides hydatid cysts into five types (I–V) [7] and the WHO Informal Working Group on Echinococcosis (WHO-IWGE) classification, which stratifies cysts into active, transitional and inactive stages (CE1–CE5) to guide clinical decisions [8]. Both systems play a critical role in diagnostic evaluation, staging and selecting appropriate therapeutic strategies—including watchful waiting, antiparasitic therapy, PAIR (puncture, aspiration, injection and re-aspiration), or surgery—particularly in complex scenarios such as pregnancy. Although PAIR is mentioned as a therapeutic option in the general discussion of echinococcosis management, it was not applied in our patient’s case due to the cyst characteristics and pregnancy status.

Hydatid cysts can be found accidentally and asymptomatic in 70% of cases [5]. Mass effect causes non-specific symptoms such abdominal pain, distension, nausea, early satiety and hematologic alterations [3]. Large cysts may cause left shoulder pain or kidney compression, causing proteinuria or hypertension. Rupture affects 4.5% of pregnant women and can cause 70% perinatal mortality, with fetal results worse than mother outcomes [9].

Hydatid disease’s vague symptoms may coincide with other symptoms of pregnancy such as nausea, vomiting and abdominal pain, making diagnosis difficult [9]. The illness may be undiagnosed until obstetric difficulties occur. Symptoms are caused by mass effect on neighboring structures or consequences such cyst rupture, biliary communication, or subsequent bacterial infection [10]. In our case report, the patient had abdominal pain and discomfort repeatedly.

**Figure 2:**
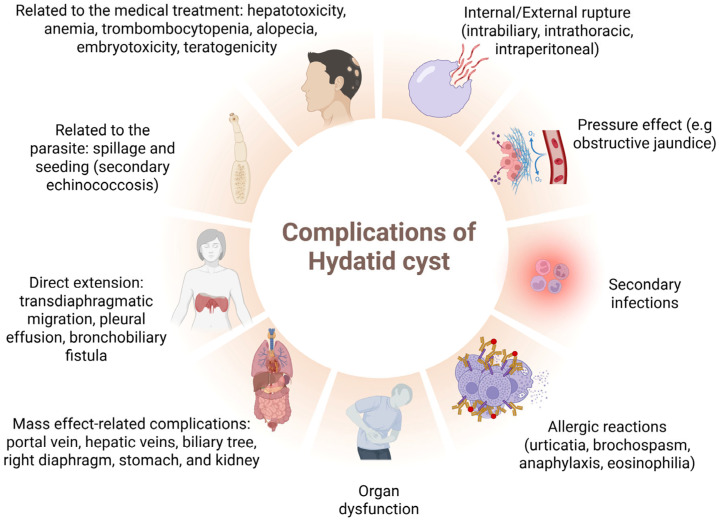
Complications of medical treatment-related hydatid cyst, clinical spectrum, including the following. Toxic effects from antiparasitic therapy (e.g., hepatotoxicity, anemia, alopecia, embryotoxicity). Parasitic factors: spillage and seeding leading to secondary echinococcosis during cyst rupture or manipulation. Structural damage via direct extension: spread through the diaphragm may result in pleural effusion, bronchobiliary fistula and other thoracic complications. Mass effect: compression of nearby structures such as the portal vein, biliary system, stomach and kidney, leading to significant dysfunction. Organ dysfunction: due to space-occupying or inflammatory effects. Allergic reactions: severe immune responses including urticaria, anaphylaxis, bronchospasm and eosinophilia. Secondary infections: cysts can become infected, leading to abscess formation and sepsis. Pressure effects: e.g., obstructive jaundice when biliary ducts are compressed. Rupture: either internal (into biliary, thoracic, or peritoneal spaces) or external, resulting in severe clinical deterioration.

As I can be seen in Figure 2, hepatic hydatid cysts can cause mechanical obstruction, dystocia, prolonged labor, spontaneous rupture, anaphylactic shock, peritoneal parasite dissemination and uterine compression by large cysts, which can cause pre-term labor or pregnancy loss [11]. The risk of rupture of the two cysts containing active daughter vesicles was our patient’s biggest concern, given their size. As pregnancy progresses, uterine volume increases intra-abdominal pressure, increasing the risk of cyst rupture and parasite spread throughout the mother and fetus [12].

Hydatid disease during pregnancy is diagnosed using imaging and serological markers. Ultrasound is favored during pregnancy due to its safety, displaying uniloculated or multiloculated cystic formations with daughter vesicles. MRI provides superior soft tissue contrast without ionizing radiation, making it suitable for anatomical delineation in some cases [13]. IgG and IgM testing for Echinococcus granulosus help confirm the diagnosis; however, their sensitivity and specificity vary [14,15].

In the inflammatory response, vascular adhesion protein-1 (VAP-1) binds leukocytes to the vascular endothelium independently of its enzymatic activity [16]. This binding is essential for leukocyte extravasation and appropriate immunological and inflammatory responses [17].

Hydatid illness in pregnancy has no official treatment standards, making it controversial [3]. Treatment must be tailored to gestational age, cyst features and maternal–fetal risk factors.

Expectant management of small, asymptomatic cysts requires close monitoring with serial imaging and serological assessments, which was not possible in our case, except for the third retroperitoneal cyst, which did not pose an immediate threat and was recommended by the surgical team to be removed postpartum [18]. Albendazole (ABZ) is a first-line antiparasitic, but its use during pregnancy is controversial due to its teratogenicity, especially in the first trimester [19]. In some circumstances, percutaneous aspiration (the PAIR technique—puncture, aspiration, scolicidal agent injection and re-aspiration) may be used, but its safety during pregnancy is unknown [20]. It is recommended for big cysts at risk of rupture, severe symptoms, or biliary or peritoneal communication [21]. However, surgery during pregnancy might cause preterm labor, hemorrhage and anaphylaxis, requiring a multidisciplinary approach [22].

## 2. Clinical Case

In April 2024, a 29-year-old woman in her third pregnancy with two previous deliveries, a non-smoker and non-drinker with a normal BMI range, at 22 weeks of gestation, with a history of a prior caesarean section and unknown history of chronic illnesses or significant medical conditions was admitted to the Emergency Department of “Spitalul Clinic de Urgenţa Sfantul Apostol Andrei” due to abdominal discomfort. She was febrile but otherwise healthy on clinical assessment. Her gravid uterus caused abdominal distension without painful contractions. Normal uterine tone, vigorous fetal movements and heartbeats were found. Normal heart rate, bowel transit and urination characterized the normotensive patient. For her symptoms, she was admitted for specialized evaluation. 

In this case, diagnostic imaging was performed using a Samsung ultrasound system, manufactured in South Korea by Samsung Medison, a leading medical device subsidiary of Samsung Electronics. The laparoscopic intervention was conducted utilizing instruments produced by Karl Storz, a German company renowned for its precision endoscopic systems, with manufacturing headquartered in Tuttlingen, Germany.

Laboratory Investigations Overview

The patient’s laboratory workup revealed generally preserved hepatic and renal function, with liver enzymes within normal limits (ALT 12 U/L, AST 14 U/L) and total bilirubin at 0.26 mg/dL. Inflammatory markers were moderately elevated, including a C-reactive protein (CRP) level of 13.4 mg/L, reflecting an active inflammatory state consistent with the diagnosis of hepatic hydatid disease. The complete blood count showed leukocytosis (WBC 12.4 × 10^9^/L), mild neutrophilia and eosinophilia (eosinophils 15.2%), a finding often associated with parasitic infections such as echinococcosis. Hemoglobin was mildly reduced at 11.5 g/dL, with normocytic indices. Coagulation parameters, including prothrombin time (PT) and activated partial thromboplastin time (aPTT), remained within normal ranges, supporting the patient’s readiness for surgical intervention.

Serologic and infectious results

Serological testing for echinococcosis demonstrated a positive IgG antibody response to *Echinococcus granulosus* (IgG 13.21 U/mL), supporting the clinical and imaging-based diagnosis. Vaginal culture results revealed mixed bacterial flora including *Streptococcus agalactiae*, without signs of acute infection. Renal function was preserved, with urea and creatinine levels within reference limits. Urinalysis showed mild leukocyturia (25 cells/μL) and isolated red blood cells (1–5/field), with no proteinuria or glycosuria, findings likely incidental in the context of pregnancy. Taken together, these results provided biochemical and immunological confirmation of the parasitic etiology and ruled out significant systemic complications, allowing the care team to proceed with definitive surgical management in the second trimester.

Imaging results

Multiple hepatic hydatid cysts were seen after imaging. A general surgery, infectious diseases and anesthesiology consultation was requested. The conclusion was that hepatic cysts posed a major maternal–fetal risk, potentially causing serious problems or death. The patient was taken to General Surgery.

**Figure 3:**
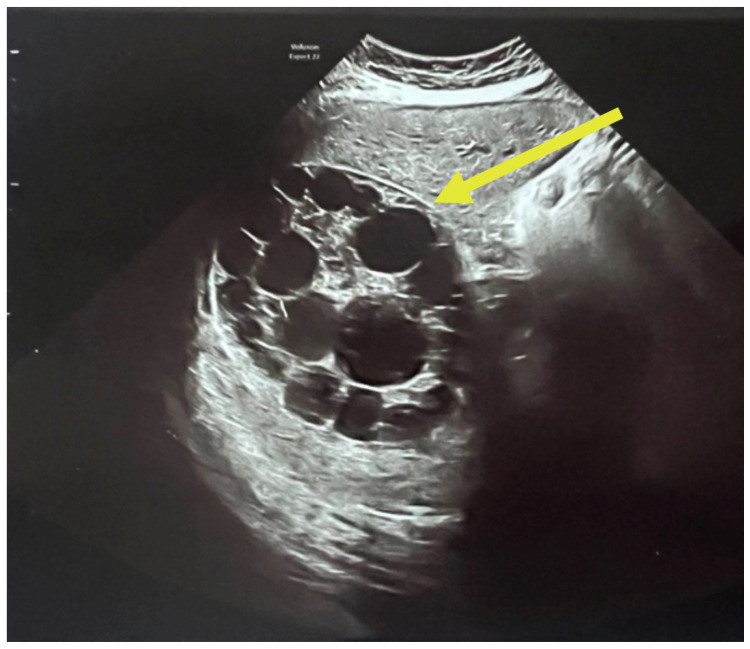
Ultrasound of hydatid cyst with multiple daughter cysts: large, well-defined hydatid cyst with internal multiple daughter vesicles—a hallmark of *Echinococcus granulosus* infection. The cyst shows a “rosette” or “honeycomb” appearance, characteristic of CE2-stage cysts per WHO classification [23].

A complete abdominal examination showed an enlarged abdomen due to the gravid uterus, movement upon respiration, slight upper abdominal quadrant pain and no peritoneal irritation. The infectious disease consultation proposed an echinococcal etiology based on cyst features and recommended serological tests for Echinococcus granulosus IgM and IgG and an abdominal and pelvic MRI. As it can be seen in Figure 3, the patient had two large hepatic hydatid cysts: a 12 × 9 cm type 2 cyst (with daughter vesicles) in the right lobe and an 8.5 × 8 cm type 4 cyst (pseudoparenchymatous) in the left lobe.

**Figure 4:**
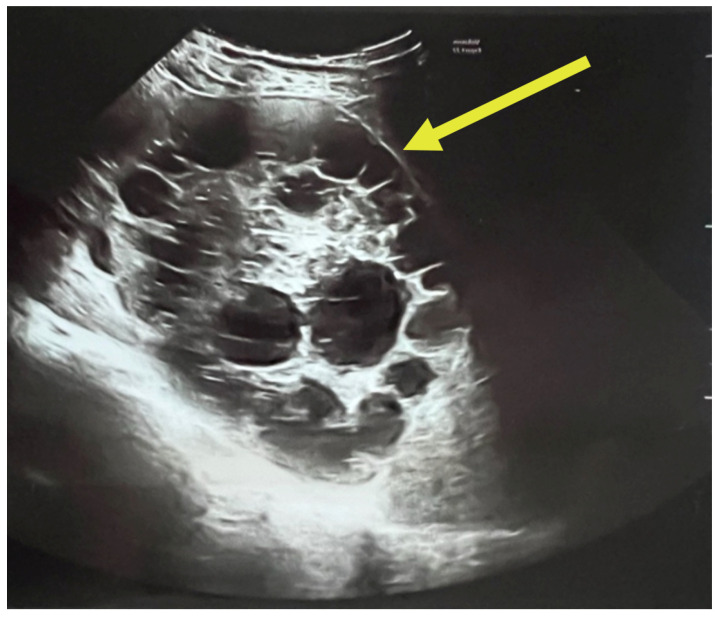
Ultrasound of the hydatid cyst with internal septations and vesicles: numerous daughter vesicles of varying sizes are visualized, separated by fine septations, enhancing the suspicion of active parasitic proliferation.

The multiplicity and defined arrangement of daughter cysts within the mother cyst reflect a high risk for spontaneous or traumatic rupture, especially concerning during pregnancy, due to the potential for anaphylaxis, secondary echinococcosis and disseminated disease. Figure 4 below shows the internal septations and vesicles of the cyst.

Normally, preoperative treatment includes albendazole 400 mg every 12 h for 28 days, followed by a 14-day break, for five cycles.

Despite infectious disease consultation recommending antiparasitic therapy with albendazole due to the large diameter of the hepatic hydatid cyst, the patient opted against initiating treatment. The decision was based on her concern about potential teratogenic risks and complications associated with albendazole exposure during pregnancy, particularly in the second trimester. Although albendazole is sometimes considered when the maternal benefit outweighs fetal risk, in this case, the patient’s preference was to avoid any pharmacological intervention that might endanger the fetus. As such, treatment was postponed until after delivery and a multidisciplinary team closely monitored the pregnancy course and cyst evolution.

Hepatoprotective medication was started for six months, with complete blood count; C-reactive protein, liver and renal function tests; and abdominal ultrasound every three weeks.

Laparoscopic surgery was chosen due to the rising danger of cyst rupture and dispersion. One week later, the patient had cystotomy with parasite evacuation, atypical hepatectomy of segments II-III, cystectomy with partial pericystectomy of segment IVa, lavage and numerous drainage placements.

The surgical field was sterile and properly draped, with a visible midline or infraumbilical port insertion site, suggesting entry for camera or primary instrument access. This method allows for reduced postoperative pain, faster recovery and lower risk of cyst rupture and dissemination, which are particularly critical during pregnancy. The procedure also aims to prevent complications like cyst rupture, anaphylaxis, or mass effect on surrounding maternal–fetal structures.

**Figure 5:**
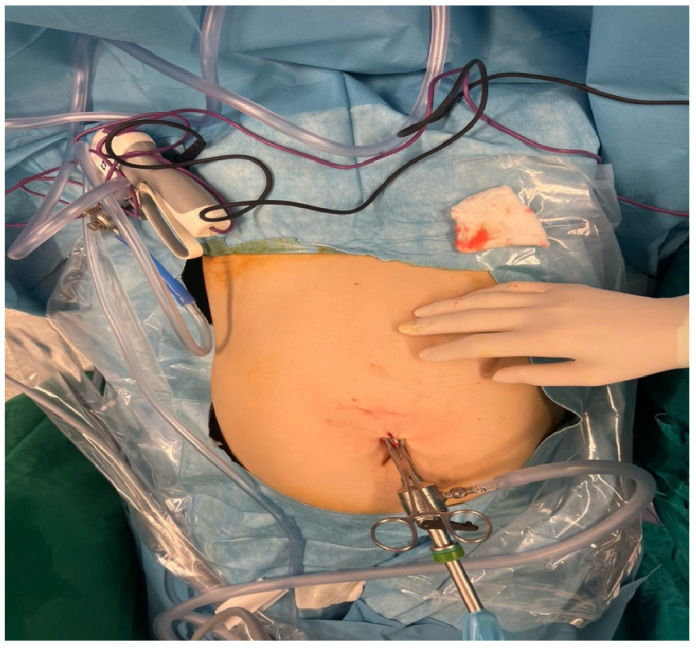
Laparoscopic intervention for hydatid cyst during pregnancy: a trocar and laparoscopic instruments are in place, with insufflation tubing and monitoring equipment.

**Figure 6:**
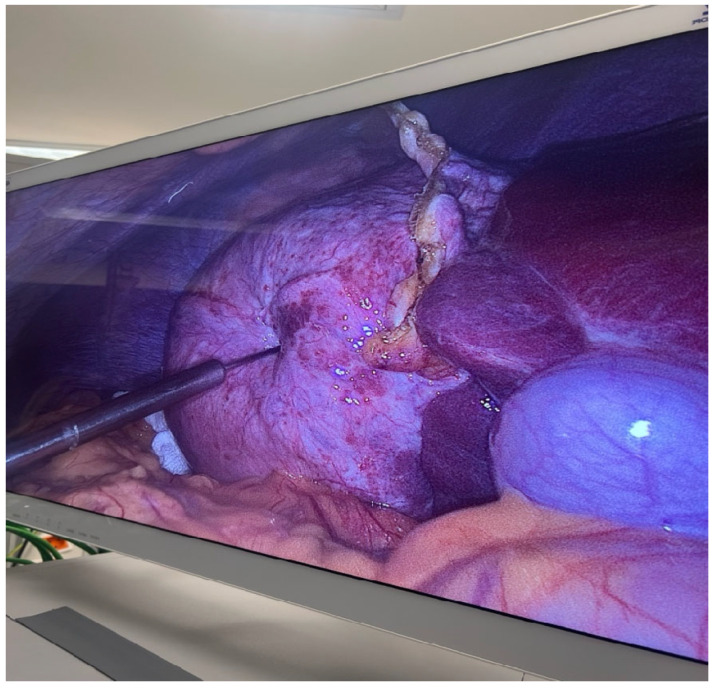
Intraoperative laparoscopic view of the hydatid cyst prior to puncture: the cyst appears as a large, tense, dome-shaped lesion on the liver surface, with visible vascular markings and surrounding adhesions; the laparoscopic instrument is carefully positioned and directed toward the cyst wall, indicating the next surgical step—controlled puncture and aspiration.

This step is crucial in the PAIRS technique, often used to decompress the cyst, reduce pressure and minimize the risk of spillage or rupture, which could lead to secondary echinococcosis or anaphylactic shock (Figure 5). The surrounding anatomy, including bowel loops and the hepatic capsule, is clearly visible in Figure 6.

This critical intraoperative moment in managing hydatid disease laparoscopically combines advanced surgical skill with stringent parasitological control measures.

**Figure 7:**
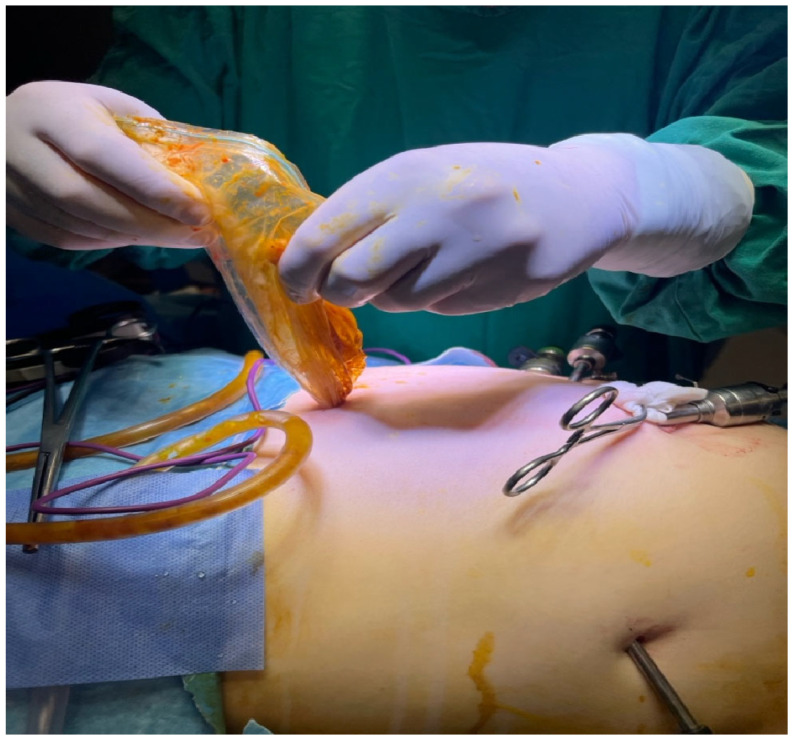
Extraction of the hydatid cyst contents via endobag during the laparoscopic surgery: the transparent endobag is being gently removed through a laparoscopic port site, with cyst contents securely enclosed to prevent contamination of the peritoneal cavity.

Visible instruments include trocars and graspers, while the abdominal cavity remains distended from pneumoperitoneum (Figure 7). The surrounding area is well-prepared and covered with sterile drapes, with residual povidone–iodine staining.

This step is a hallmark of safe parasitic surgery, especially crucial in pregnant patients or immunocompromised individuals, ensuring that parasite containment is maintained from intraoperative manipulation through specimen extraction.

**Figure 8:**
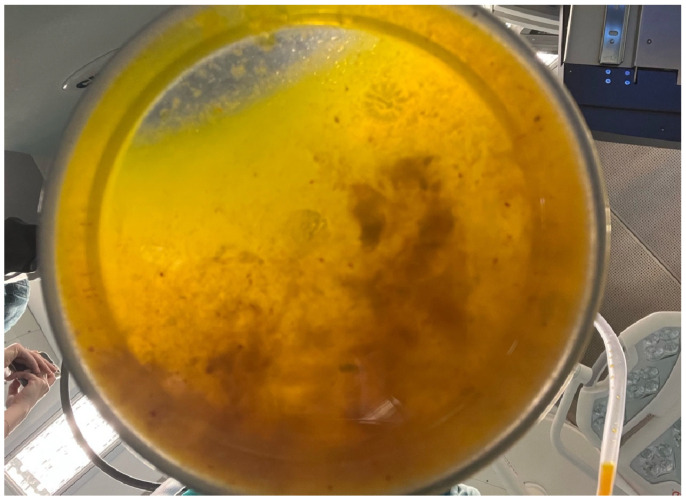
Aspirated hydatid cyst fluid—postoperative collection: the fluid appears dense and particulate, consistent with the presence of protoscolices, hydatid sand and laminated membrane debris, hallmark features of *Echinococcus granulosus* cystic content.

As can be seen in Figure 8, the material was collected under strict aseptic conditions during laparoscopic aspiration. This visual represents a successful drainage procedure, essential to minimize the risk of rupture, secondary echinococcosis and anaphylactic reactions.

**Figure 9:**
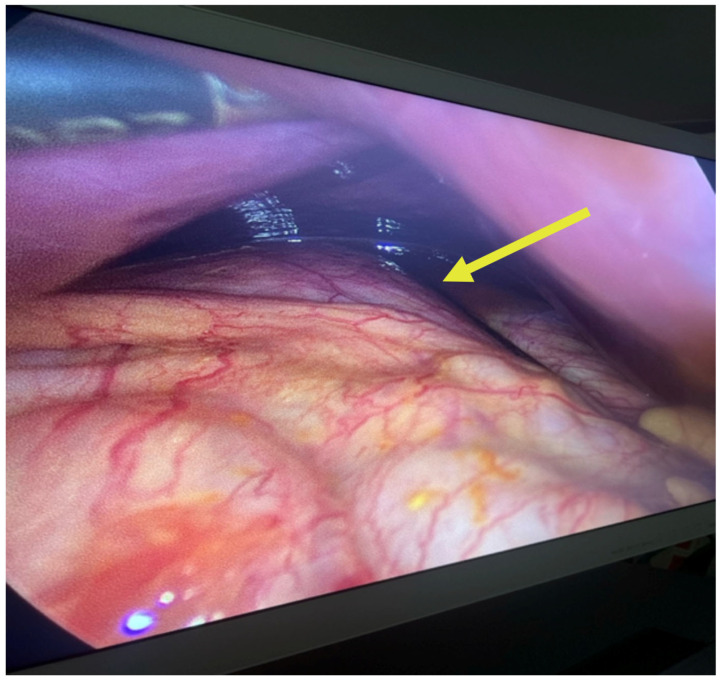
Intraoperative view of third cystic lesion with suspected renal origin: a third cystic structure was observed intraoperatively, with a suspected but unconfirmed renal origin. The cyst appears tense, well encapsulated and partially retroperitoneal, nestled deep within the surgical field. Prominent surface vasculature is visible, traversing a glistening cyst wall with adjacent peritoneal reflections and intestinal loops.

Intraoperatively, an additional abdominal ultrasound revealed a third hydatid cyst measuring 12 × 9 cm, located in the right flank, retroperitoneally under the liver. Due to its positioning, the risk of rupture was deemed minimal and it was decided to delay its evacuation until after delivery to ensure maternal and fetal safety (Figure 9).

The anatomical orientation and visual context suggest possible proximity to the posterior abdominal wall, raising suspicion of renal involvement. However, due to overlapping features, differential diagnoses remain broad, including origins from retroperitoneal tissue, adrenal gland, or mesenteric structures. However, there is no clear demarcation from adjacent organs and there is a lack of confirmatory imaging at this surgical stage.

The anatomical depth and positioning seen on the ultrasound raise strong suspicion that the lesion may originate from the kidney, although the exact organ of origin remains uncertain intraoperatively. Differential considerations include cystic lesions (CLs) of retroperitoneal, adrenal, or mesenteric origin.

Postoperatively, the patient received antibiotics, analgesia and fluid–electrolyte rebalancing. No fetal distress or uterine contractions occurred. The small stomach drainage was evacuated and the patient was released well. A long-term follow-up was planned to assess cyst progression and maternal–fetal health.

**Figure 10:**
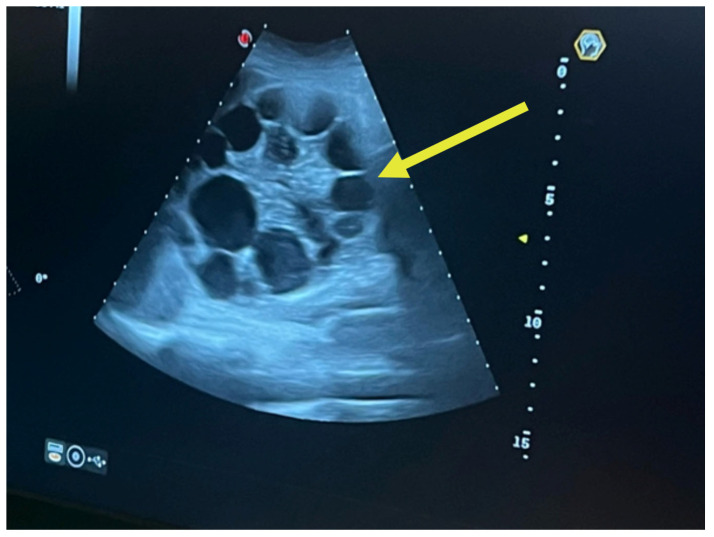
Intraoperative ultrasound revealing suspected renal-origin hydatid cyst (third lesion): the scan reveals a complex, multiloculated cystic structure with multiple anechoic (black) daughter cysts contained within a well-defined parent cyst.

The patient reported abdominal pain to emergency medical personnel periodically. An abdominal and pelvic scan after another hospital visit in July 2024 revealed the following: the liver, pancreas and spleen were normal in size, structure and echogenicity. A normal gallbladder, thin walls and no calculi were observed. Multiple cysts filled the right kidney. A normal-sized left kidney with maintained parenchymal structure and no calculi was observed. A normal-sized urinary bladder contained anechoic fluid. The uterus showed pregnancy, with fetal cardiac activity and no peritoneal fluid. Obstetric ultrasound revealed 34 weeks of gestation, posteriorly implanted placenta, normal amniotic fluid index and estimated fetal weight of 1803 g ± 10% (Figure 10).

The patient’s abdominal pain prompted a surgical consultation, which diagnosed abdominal meteorism. Espumisan (simethicone) pills, two per day, were recommended pending obstetrician approval. Antispasmodic treatment improved the patient’s symptoms and she was discharged in stable condition with afebrile status, a well-distended abdomen due to the gravid uterus, no painful uterine contractions, normal basal uterine tone, fetal heart activity and active fetal movements, no vaginal bleeding or amniotic fluid leakage, preserved intestinal transit for gas and stool and normal micturition.

On 8 August 2024, at 38–39 weeks of gestation, the patient underwent a cesarean section, delivering a live female neonate weighing 2680 g, with an Apgar score of 9. The surgical indications included the following: G3P2 (III G, II P), singleton pregnancy, intact membranes, cephalic presentation, scarred uterus with imminent uterine rupture, active labor.

During the cesarean section, a bilateral tubal ligation was performed at the patient’s request. The postoperative evolution was favorable, with management including antispasmodic, uterotonic, anticoagulant, anti-inflammatory, analgesic, prokinetic and fluid-electrolyte rebalancing therapy. Sterile cervical cultures ruled out postpartum infections. Follow-up hematology evaluation recommended post-treatment for anemia.

Following strict adherence to the general surgery recommendations, the patient underwent an abdominal and pelvic MRI with contrast four months postpartum. The imaging findings confirmed the diagnosis of a large retroperitoneal hydatid cyst in the right flank, located subhepatically, containing daughter vesicles. The cyst was most likely of renal origin, classified as CE3b under the WHO-IWGE classification [8].

The World Health Organization revised the classification of cystic echinococcosis to reflect its natural progression [8]. Initially simple and undifferentiated, these cysts are labeled as “cystic lesions” due to uncertain origin. CE Types 1 and 2 are considered active and fertile, containing viable protoscoleces. Type 3 cysts represent a transitional phase with partial structural compromise. Types 4 and 5 are inactive, degenerative cysts that have typically lost fertility [24].

Serial laboratory tests showed moderate anemia, with hemoglobin levels averaging 8.7 g/dL, decreasing C-reactive protein levels (from 2.18 to 0.69 mg/dl), normal blood glucose levels, unremarkable urinalysis, no infection and negative HIV and RPR serology. Her blood group is A(II), Rh-positive. Table 1 below shows the classification of hydatid cyst.

Our patient presented at 22 weeks of gestation with multiple hepatic hydatid cysts, one of which extended retroperitoneally and was suspected to be of renal origin. Notably, the pregnancy was preserved and the surgical intervention was successfully performed laparoscopically without fetal compromise. The third cyst, being asymptomatic and low-risk, was deferred for postpartum management. Delivery occurred at term via cesarean section, with both maternal and neonatal outcomes favorable and definitive imaging and follow-up were completed postpartum.

From an obstetrical perspective, the uterus was noted to be anteverted, of normal dimensions, with a clear uterine cavity and no focal abnormalities. An isthmic anterior cesarean scar was present with no pathological findings.

In February 2025, a second laparoscopic procedure was performed to address the retroperitoneal hydatid cyst suspected to originate from the right kidney, initially left in situ during the antenatal surgical intervention due to its low rupture risk and stable clinical profile. The cyst was successfully excised using a minimally invasive approach, without intraoperative complications and with particular attention to preventing spillage of parasitic material, given the inherent risk of peritoneal contamination.

When compared to the three cases reported between 2008 and 2010 involving renal hydatid disease, several points of convergence and divergence become apparent. In the referenced series, all patients had left renal involvement, with one case demonstrating hepatic co-involvement, similar to our patient. The predominant symptomatology was abdominal pain and flank mass and diagnosis was consistently established through abdominal ultrasonography and CT imaging [26].

In our case report, the decision to delay the renal cyst excision until postpartum allowed for a safer surgical environment, free from the anatomical and physiological constraints imposed by the gravid uterus.

Histopathological Examination

**Figure 11:**
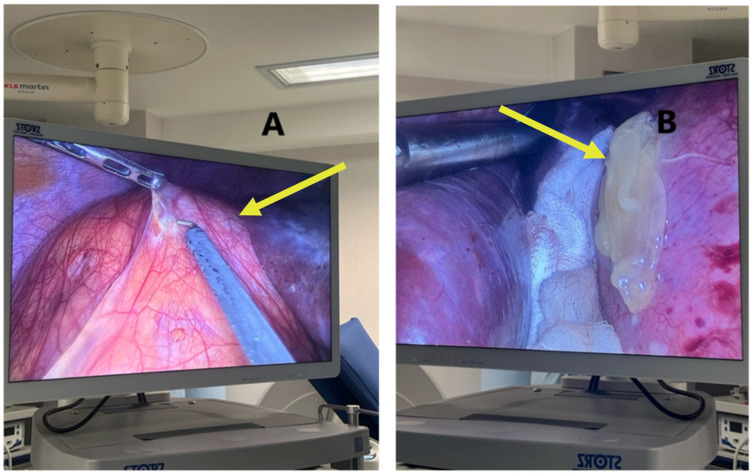
Puncture and controlled evacuation of suspected renal hydatid cyst: (**A**)—precise puncture of the cyst wall using a laparoscopic aspiration needle, while graspers maintain tension on adjacent tissue to prevent spillage; (**B**)—the exteriorization of the cyst contents, including hydatid membranes and daughter vesicles, with a macroscopic appearance remarkably similar to the previously removed hepatic hydatid cysts. The yellowish, gelatinous material confirms the parasitic nature of the lesion.

Histopathological analysis remains a critical component in confirming the diagnosis of hydatid cysts and differentiating them from other cystic hepatic or renal lesions, particularly in complex or atypical cases. In the presented case, surgical specimens obtained during laparoscopic cystectomy were submitted for pathological evaluation, including both the hepatic cysts and, postpartum, the retroperitoneal cyst suspected to originate from the right kidney. Figure 11 shoes the Puncture and controlled evacuation of suspected renal hydatid cyst.

**Figure 12:**
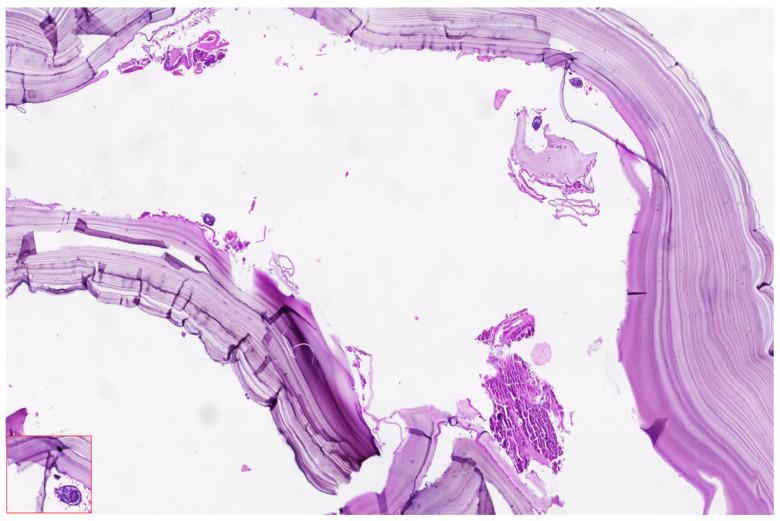
Histopathological appearance of hydatid cyst wall showing laminated membrane and germinal layer.

Figure 12 shows the histopathological appearance of hydatid cyst.

Microscopic examination of the cyst wall revealed the characteristic trilaminar structure typical of Echinococcus granulosus infection. The outermost layer consisted of an acellular laminated membrane, eosinophilic in nature, which is secreted by the parasite and serves as a physical and immunologic barrier. Beneath this layer was the germinal membrane, composed of nucleated cells that give rise to brood capsules and protoscolices. Notably, protoscolices (indicative of cyst fertility) were identified in clusters, exhibiting typical hooklets and sucker structures when stained with hematoxylin and eosin, particularly at magnifications of 20× and 40×.

The aspirated cyst fluid, rich in hydatid sand, contained free hooklets, degenerating protoscolices and debris from laminated membrane fragments. These findings confirmed the diagnosis of active, fertile cysts classified as WHO-IWGE CE2 and CE3b types, respectively, consistent with the imaging profile and intraoperative appearance.

There was no evidence of secondary infection, malignancy, or necrosis in the examined specimens. The inflammatory infiltrate surrounding the cysts included eosinophils, lymphocytes and occasional multinucleated giant cells, consistent with a chronic immune response to the parasitic infestation.

Postoperative blood results

**Figure 13:**
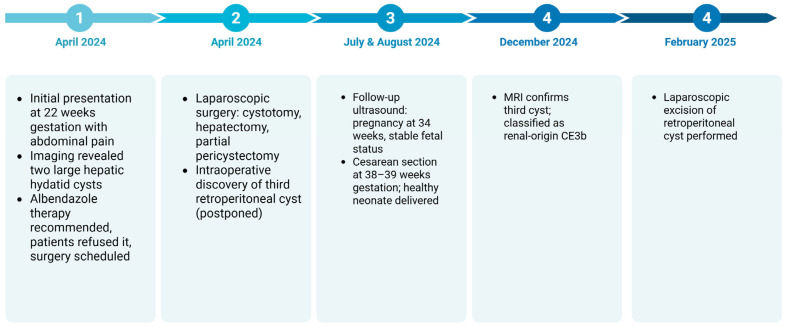
Chronological timeline of diagnosis, treatment and pregnancy outcome.

The postoperative laboratory evaluation showed stable biochemical and hematological parameters, indicating favorable recovery following surgical intervention. Liver function tests remained within normal limits (ALT 10 U/L, AST 11 U/L), suggesting no hepatic injury post-hepatectomy. Inflammatory markers showed a slight elevation in CRP levels, measured at 21.4 mg/L, which was expected in the early postoperative period and not indicative of infection. Figure 13 shows the timeline of events for this patient.

It can be seen that close multidisciplinary monitoring ensured favorable outcomes for both mother and fetus, with a healthy cesarean delivery at term and successful postpartum cyst removal.

## 3. Discussion

Hydatid disease is caused by larval forms of cestodes belonging to the genus Echinococcus, of which two species are of primary clinical significance: Echinococcus granulosus, the causative agent of cystic echinococcosis and Echinococcus multilocularis, responsible for alveolar echinococcosis [27].

The immunosuppressive state of pregnancy, particularly the decrease in cell-mediated immunity, may facilitate accelerated growth and expansion of hydatid cysts, increasing the risk of rupture, infection, or peritoneal dissemination, particularly in cases involving large or multivesicular cysts [28].

To contextualize our case and highlight the variability in clinical presentation and management of echinococcosis during pregnancy, we reviewed published case reports from both the European and global literature. The following table summarizes 15 representative cases, outlining the gestational timing, cyst location and clinical course. These reports underscore the heterogeneity of hydatid disease in pregnant women, ranging from asymptomatic incidental findings to life-threatening complications requiring urgent intervention. This comparative overview emphasizes the importance of individualized treatment strategies and supports the need for multidisciplinary decision-making in managing such rare and complex cases (Table 2).

A comparative reflection between our case and the one reported by Bhirud et al. [41] highlights both similar clinical challenges and divergent therapeutic pathways, shaped by gestational age, cyst location and patient-specific risk considerations.

In the referenced case [41], a 36-year-old patient was diagnosed during the first trimester with symptomatic renal hydatid disease and management involved medical termination of pregnancy, followed by robot-assisted laparoscopic pericystectomy. This decision was made following multidisciplinary counseling, weighing the maternal risks of surgical intervention during early gestation against fetal viability and the potential for complications if surgery were delayed.

From a diagnostic standpoint, ultrasound is the primary modality used, consistent with safety considerations in pregnancy [42]. While serological tests (ELISA, IHA, CF) are discussed extensively in the literature and can aid diagnosis and follow-up, in our patient, imaging played the central role, with MRI utilized postpartum for comprehensive anatomical assessment [43].

We also found similarities to a South African patient who presented at 7 weeks of gestation with abdominal pain and fever and was diagnosed with a hepatic hydatid cyst by ultrasound [44]. Like our patient, imaging confirmed the diagnosis and both women had caesarean sections at term with good maternal and neonatal outcomes and no postpartum complications. Our patient did not have fever, but stomach pain triggered medical investigation, which revealed the hydatid cysts. Early imaging and interdisciplinary monitoring are crucial for controlling rare parasite infections during pregnancy, as shown by the ultrasound identification of hepatic hydatidosis and the successful, complication-free births [45].

Therapeutically, both scenarios acknowledge the three main treatment pathways: medical therapy, PAIR and surgical intervention [46]. In the referenced literature, albendazole is described as the cornerstone of medical therapy, though classified as FDA pregnancy category C, due to its teratogenic potential, especially in the first trimester. In our case, albendazole was initiated cautiously, but given the large cyst volume and high rupture risk, medical management alone was deemed insufficient [47].

One of the primary therapeutic challenges in managing hydatid disease during pregnancy lies in selecting the appropriate treatment strategy, which largely depends on the sonographic classification of the cyst [48]. According to Khazaal et al. [49], Gharbi type I and II cysts are generally amenable to ultrasound-guided percutaneous aspiration techniques such as PAIR. In contrast, type III and IV cysts, excluding those with extensive calcification, tend to necessitate surgical intervention [49].

In our case, the decision to defer surgical removal of the third hydatid cyst, located retroperitoneally under the liver, until after delivery was based on its anatomical positioning and clinical behavior. Unlike the hepatic cysts, this third lesion demonstrated a lower risk of rupture due to its encapsulated structure, absence of active compression symptoms and limited vascular proximity. Given these factors and to minimize fetal exposure to anesthesia, surgical stress and potential intraoperative complications, the multidisciplinary team opted for conservative management during pregnancy. The cyst remained stable throughout the gestational period and was successfully excised postpartum under controlled laparoscopic conditions, ensuring both maternal safety and neonatal viability.

Comparing our case to two similar clinical studies shows noteworthy similarities and differences in hepatic hydatid cyst presentation and management during pregnancy [32]. All three patients had significant hydatid cysts diagnosed by ultrasonography, either due to stomach pain or incidentally, despite their different obstetric histories and gestational ages.

In the first case, a 28-year-old primigravida at 13 weeks of gestation reported with right upper quadrant pain. The big hydatid cyst (137 × 190 × 203 mm) in hepatic segments V and VI was surgically handled with partial cystectomy [50]. The procedure was uneventful, with a full-term vaginal delivery of a healthy infant and no further echinococcal spread.

In the second patient, a 31-year-old multigravida, a 160 × 150 × 105 mm hepatic cyst was verified by ultrasound and MRI at 23 weeks. Postoperative biliary drainage cleared spontaneously after her partial cystectomy. A healthy newborn was delivered at 34 weeks without maternal illness worsening [50].

Our case presents the unique complexity of multiple hepatic hydatid cysts, diagnosed at 22 weeks in a multigravida with a scarred uterus. Unlike the aforementioned cases, the patient exhibited no febrile episodes, but did report recurrent abdominal discomfort, raising clinical concern. What distinguishes our scenario is the multifocal cystic involvement, including a third retroperitoneal cyst presumed to be of renal origin and the decision for laparoscopic surgical management—a less invasive yet highly effective approach. The hydatid cysts displayed daughter vesicles and marked enlargement, posing a substantial risk of rupture and peritoneal dissemination, especially with advancing gestation and increasing intra-abdominal pressure.

While the patients in the comparative cases underwent open subcostal surgeries, our patient benefited from a minimally invasive approach, followed by close antenatal monitoring and elective cesarean delivery at term. Moreover, all three patients successfully delivered live neonates with favorable maternal and neonatal outcomes, underscoring the importance of early imaging, individualized risk stratification and interdisciplinary collaboration in managing hydatid disease during pregnancy.

The case reported by Javanbakht et al. [51] tragically illustrates the potential lethality of percutaneous treatment in hydatid disease during pregnancy, particularly when complicated by anaphylactic shock following cyst rupture. Despite the initial hemodynamic stability of the patient and appropriate monitoring, the PAIR procedure resulted in acute respiratory and cardiovascular collapse, necessitating extensive resuscitation efforts [51]. The patient developed severe metabolic acidosis, experienced multiple cardiac arrests and ultimately died from what was presumed to be a systemic anaphylactic reaction to parasitic fluid spillage—a rare but often fatal event in the context of echinococcosis during gestation [51].

In contrast, our patient underwent a carefully planned laparoscopic surgical procedure during the second trimester, a period widely recognized as the safest window for operative interventions in pregnancy. The decision to choose laparoscopy over percutaneous drainage was made based on the cyst characteristics, specifically, the presence of daughter vesicles, significant cyst volume and an elevated risk of spontaneous rupture—factors that rendered conservative or minimally invasive approaches suboptimal in this case.

The surgical team implemented a well-structured intraoperative protocol, which included real-time ultrasound guidance, controlled aspiration and safe extraction of the cyst using an endobag system to prevent peritoneal contamination. Throughout the procedure, the patient maintained stable vital signs and no intraoperative or postoperative complications were observed. The pregnancy subsequently evolved without incident, culminating in the delivery of a healthy term neonate by cesarean section.

Experimental studies have demonstrated that albendazole can exert embryotoxic effects, particularly in rodent models [12,52]. In vitro assays have shown that ABZ significantly impairs differentiation of mesencephalic and limb bud cells derived from rat embryos, even at concentrations that do not induce overt cytotoxicity. Furthermore, in vivo studies in rats have established that ABZ exhibits teratogenicity at dose levels that do not cause maternal toxicity. The lowest observed effect level (LOEL) and no observed effect level (NOEL) have been identified as 6.62 mg/kg and 5 mg/kg body weight/day.

The teratogenic effects of ABZ are primarily attributed to its active metabolite, albendazole sulfoxide (ABZ-SO), which achieves higher and more prolonged plasma concentrations compared to its parent compound [29]. Among the various congenital anomalies reported, limb malformations appear to be the most sensitive developmental endpoint, highlighting the significant risks associated with albendazole exposure during gestation [53].

When compared to the patient cohort in which albendazole therapy was initiated in six pregnant women with hydatid disease, our case shares important similarities while also highlighting a distinct clinical trajectory [54]. In the referenced group, percutaneous drainage was employed in one case, whereas half of the patients who initially received medical treatment required subsequent surgical intervention due to the emergence of maternal complications. This underscores the potential limitations of conservative therapy in cases where the cyst burden is high or where disease progression compromises maternal health.

Similarly, in our case, although albendazole was initiated as part of the management plan, the presence of two voluminous hepatic cysts with daughter vesicles and high rupture risk necessitated timely surgical intervention during pregnancy, rather than relying solely on pharmacologic control. Notably, while two patients from the comparative study delivered via cesarean section for obstetric reasons, our patient underwent a planned cesarean delivery at term, following close antenatal monitoring and postpartum surgical planning for the residual retroperitoneal cyst [43].

Although the teratogenic potential of albendazole at full-term gestation is well documented, the precise pathophysiological mechanisms underlying these effects remain unclear. Currently, there is a lack of comprehensive data regarding the specific processes leading to fetal malformations, both in vitro and in vivo [54]. To bridge this knowledge gap, ABZ has been selected as a model compound for an in vivo study aimed at investigating the developmental toxicity induced in rat embryos during early organogenesis (gestational days 10–12) [55]. Additionally, gestational day (GD) has been designated as a critical observational window to examine the early onset of structural anomalies detected in full-term rat fetuses [56].

Although our patient experienced a favorable maternal and fetal outcome following surgical intervention, it is important to recognize that the pathophysiological impact of Echinococcus granulosus infection during pregnancy may extend beyond the immediately observable clinical picture.

A notable example comes from an animal study using BALB/c mice experimentally infected with Echinococcus granulosus [9]. The findings demonstrated that maternal infection significantly compromised pregnancy outcomes. Three out of ten infected mice did not conceive and those that did had fewer offspring with reduced size and weight compared to controls. In addition, hormonal imbalances were observed, including decreased progesterone and increased estradiol levels, pointing to a potential disruption in reproductive physiology caused by the parasitic infection.

## 4. Limitations

Despite these general guidelines, the lack of robust data and prospective trials has led to considerable variation in clinical practice and no universally accepted management protocol currently exists for hydatid disease in pregnant patients.

To address this gap, an evidence-based algorithm, derived from the only meta-analysis to date on this topic [47], has been proposed to guide clinical decision-making in pregnancy complicated by hydatid disease This algorithm integrates gestational age, cyst type and maternal–fetal risk assessment to determine whether conservative, medical, percutaneous, or surgical management is most appropriate.

## 5. Conclusions

Although uncommon, hydatid disease can develop during pregnancy, particularly in regions where the condition is endemic.

Diagnosing hydatid disease in pregnant patients necessitates a heightened level of clinical suspicion. Hepatic hydatid cysts during pregnancy pose significant risks to both maternal and fetal health. The management of hydatid disease in pregnancy remains a complex challenge, as there is still no universally accepted treatment protocol.

In this paper we emphasize the necessity for interdisciplinary care and long-term follow-up due to the intricate relationship between hydatid illness and pregnancy. The presence of a renal retroperitoneal hydatid cyst calls attention to the importance of postpartum imaging in parasitic-infected patients, especially in endemic areas. The successful maternal and newborn outcomes show that individualized care strategies can balance surgical risks with maternal–fetal safety. Minimizing maternal and fetal morbidity and optimizing treatment outcomes requires a multidisciplinary approach.

Timely surgical intervention during the second trimester can be both safe and effective when guided by cyst characteristics and gestational considerations. Equally critical is the role of structured postpartum follow-up, which enables definitive imaging, monitoring of residual or asymptomatic lesions and completion of staged surgical treatment when necessary.

## Figures and Tables

**Table 1:** Sonographic classification of hydatid cysts [25].

Gharbi Type	WHO Type	Cyst Morphology
I	CE 1	Unilocular anechoic lesion with double line sign
III	CE 2	Multiseptated rosette like honeycomb cyst
II	CE 3A	Cyst with detached membranes (water-lily sign)
III	CE 3B	Cyst with daughter cysts in solid matrix
IV	CE 4	Cyst with heterogeneous hypoechoic/hyperechoic contents. No daughter cysts
V	CE 5	Solid plus calcified wall

**Table 2:** Reported cases of echinococcosis during pregnancy (last 10 years, PubMed database).

Study	Origin	Trimester	Cyst Location	Treatment Approach	Clinical Case Description
Beacom et al. [29]	UK	Postpartum	Lung	Albendazole post-delivery	Isolated pulmonary hydatid cyst identified after delivery; managed medically with albendazole, with reduction in cyst size.
Li et al. [4]	China (Tibet)	Third	Pelvis and liver	Cyst drainage, vaginal delivery	Large pelvic cyst caused breech presentation; drained via posterior fornix, followed by successful vaginal delivery.
Çankaya and Yeşilyurt [30]	Turkey	Not specified	Liver	Not detailed	Rare case of giant hydatid cysts during pregnancy; specifics of intervention not detailed.
Ilhan Sezer et al. [9]	Turkey	Second	Lungs (bilateral)	Surgical management	Multiple large pulmonary cysts causing asphyxia; surgical management during pregnancy with good outcomes.
Brezeanu et al. [15]	Romania	First	Right adnexa (broad ligament)	Surgical excision, C-section	15-year-old pregnant girl with pelvic pain; adnexal hydatid cyst excised surgically, C-section due to fetal distress.
Azlin et al. [31]	Malaysia (Syrian refugee)	Not clearly stated (likely second)	Lung	Medical treatment after rupture	Pulmonary cyst rupture during pregnancy in a Syrian refugee; confirmed by sputum exam; treated promptly.
Pencovich et al. [32]	Israel	Various (17, 19, 30 weeks)	Liver	Liver resection during pregnancy	Three cases requiring major liver resection during pregnancy, including for a giant hydatid cyst; all pregnancies continued to term.
Yaman et al. [33]	Turkey	Third	Heart (interventricular septum)	Cardiac management during cesarean	Cardiac hydatid cyst causing chest pain and ventricular tachycardia during cesarean; managed successfully.
Iordanidou et al. [34]	Greece	Second	Abdomen (Morgagni cyst)	Emergency surgery	Hydatid cyst with synchronous appendicitis causing acute abdomen; surgical intervention required.
Stojkovic et al. [35]	Germany (migrant context)	Not specified	Liver	Radiological diagnosis	Case in the context of migration; hydatid cyst in liver diagnosed radiologically; included as part of series.
Bakdik et al. [36]	Turkey	First to second	Liver and spleen	Percutaneous catheter and alcohol	Multiple hepatic and splenic cysts treated percutaneously with catheter and alcohol during second trimester; no major complications.
Tolan et al. [37]	Turkey	Post-transplant/postpartum	Liver (transplanted organ)	Post-transplant observation	Pregnancy and successful birth following liver transplantation using a liver with hydatid cyst from a 93-year-old donor.
Segura-Gago et al. [38]	Peru	Third	Spleen	C-section followed by excision	Multiloculated splenic hydatid disease diagnosed late in pregnancy; C-section followed by surgical excision.
Bohîlțea et al. [39]	Romania	Not specified	Paraovarian	Laparoscopic excision	Large asymptomatic paraovarian cyst discovered on ultrasound; laparoscopic excision confirmed hydatid origin.
Llanos et al. [40]	USA (Hispanic origin)	Second	Lung	Abscess drainage and antibiotics	Pregnant woman presented with chest pain and pleural effusion; found to have a lung abscess due to hydatid disease.

## Data Availability

The original contributions presented in the study are included in the article, further inquiries can be directed to the corresponding authors.
